# Test–retest stability of patient experience items derived from the national GP patient survey

**DOI:** 10.1186/s40064-016-3377-9

**Published:** 2016-10-07

**Authors:** Antoinette F. Davey, Martin J. Roberts, Luke Mounce, Inocencio Maramba, John L. Campbell

**Affiliations:** 1 Primary Care Research Group, University of Exeter Medical School, St Lukes Campus, Exeter, EX1 2LU UK; 2 Collaboration for the Advancement of Medical Education Research and Assessment (CAMERA), Plymouth University Peninsula Schools of Medicine and Dentistry, Plymouth, UK; 3 NIHR CLAHRC South West Peninsula (PenCLAHRC), Plymouth University Peninsula Schools of Medicine and Dentistry, Room N10, ITTC Building, Plymouth Science Park, Derriford, Plymouth, Devon PL6 8BX UK

## Abstract

**Purpose:**

The validity and reliability of various items on the GP Patient Survey (GPPS) survey have been reported, however stability of patient responses over time has not been tested. The purpose of this study was to determine the test–retest reliability of the core items from the GPPS.

**Methods:**

Patients who had recently consulted participating GPs in five general practices across the South West England were sent a postal questionnaire comprising of 54 items concerning their experience of their consultation and the care they received from the GP practice. Patients returning the questionnaire within 3 weeks of mail-out were sent a second identical (retest) questionnaire. Stability of responses was assessed by raw agreement rates and Cohen’s kappa (for categorical response items) and intraclass correlation coefficients and means (for ordinal response items).

**Results:**

348 of 597 Patients returned a retest questionnaire (58.3 % response rate). In comparison to the test phase, patients responding to the retest phase were older and more likely to have white British ethnicity. Raw agreement rates for the 33 categorical items ranged from 66 to 100 % (mean 88 %) while the kappa coefficients ranged from 0.00 to 1.00 (mean 0.53). Intraclass correlation coefficients for the 21 ordinal items averaged 0.67 (range 0.44–0.77).

**Conclusions:**

Formal testing of items from the national GP patient survey examining patient experience in primary care highlighted their acceptable temporal stability several weeks following a GP consultation.

## Background

Patient surveys have been adopted widely, both in the UK and elsewhere, as a means of capturing patients’ experience of care delivered in primary and secondary care settings. Information obtained through such surveys offers the potential to inform service development and continuous quality improvement. Although offering such potential, previous research has identified concerns raised by doctors and others concerning the reliability and credibility of survey results (Asprey et al. 2013). A recent study exploring the views of primary care staff around the utility of patient experience surveys highlighted concerns regarding the perceived weakness of survey methods, the robustness of local surveys, and the rigidity of survey methodology in accurately capturing the complexity of health-care interactions (Boiko et al. 2013). A range of primary care patient experience surveys have been published which have been subjected to formal psychometric testing including some where the stability of responses over time have been documented (Wright et al. 2012; Greco et al. 1999; Mead et al. 2008; Greco and Sweeney 2003; Pettersen et al. 2004).

The English GP Patient Survey (GPPS) is a large-scale survey of patient experience of primary care routinely reported at the level of data aggregated by practice. Evidence supporting the validity and reliability of the questionnaire has already been published (Campbell et al. 2009). The questionnaire items address a range of issues relating to the accessibility of care, the quality of interpersonal care and a number of other important domains including an overall impression of patient satisfaction with care.

Although the GPPS is the largest survey of primary care undertaken in England, and having results which directly inform the NHS outcomes framework (Department of Health 2013), the stability of patient responses over time has not been tested or reported. Governance restrictions on the national GP patient survey preclude an evaluation of test–retest stability in the national data. We therefore aimed to explore this important aspect of the performance of the questionnaire using items from the national survey deployed in a postal survey in primary care.

## Methods

### Patients

Patients over the age of 18 who had attended a consultation with their general practitioner within the previous 21 day period were sent a postal questionnaire.

### Procedures

As part of a larger study examining patient’s report of their experience of care provided by general practitioners (Roberts et al. 2014), we invited five practices to take part in the test phase between November 2011 and June 2013 across the South West of England (Bristol, Devon, and Cornwall). Non-training grade GPs within participating practices who worked less than four sessions a week, locums and GPs in training were excluded from the study. Approval for the study was obtained from the South West 2 Research Ethics Committee on 28 January 2011 (ref: 09/H0202/65).

Searches were carried out on practice computer systems, generating lists of patients who had face-to-face consultations with participating doctors within a 21 day period prior to the search. Doctors screened their lists to exclude recent deaths, terminal illness, and mental incapacity. Eligible patients were posted a questionnaire pack containing a practice headed invitation letter, study information sheet, questionnaire and a prepaid return envelope. The patient information sheet provided an outline of the study; patients’ consent to participation was inferred by the return of a completed questionnaire.

Doctors within the five participating practices who had the highest initial response rates were selected by the research team to take part in the retest phase. Patients returning the test phase questionnaire within 3 weeks of mail out were sent a retest questionnaire pack. The retest questionnaire pack was identical to the test phase, except for the colour of the questionnaire. The accompanying information sheet explained why patients were receiving the second questionnaire. Returns of the retest questionnaire were accepted up to 4 weeks after their initial mail out. The gap between completion of the first (test) questionnaire and completion of the retest questionnaire could therefore vary between 3 and 49 days; the gap between the consultation and completion of the retest could vary between 30 and 76 days (Table 1).

**Table 1:**
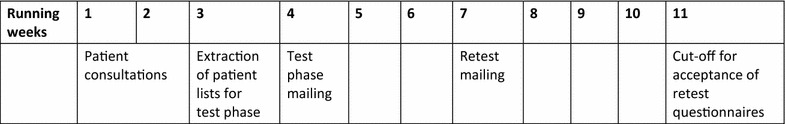
Data collection timeline for each practice, including re-test questionnaire where required

### Questionnaire items

Our questionnaire (“Appendix”) was based closely on the national GP Patient Survey (GPPS: Year 5 Q4 version). The questionnaire included questions on access, waiting times, opening hours, continuity and interpersonal aspects of care and basic socio-demographic questions, such as age, gender and ethnicity. All the questions apart from the interpersonal and continuity items were identical to the GPPS (Roberts et al. 2014). As the primary aim of the main study was to focus on patient assessment of individual doctors’ communication skills, patients were asked to complete seven items relating to inter-personal aspects of doctors care, and relating to continuity of care, referencing a consultation with a named GP on a specific date, as stated in a covering letter (Roberts et al. 2014). This context was slightly different from that of the national GPPS where patients are asked to complete these items in relation to all consultations that have occurred over the past 6 months, rather than a specific consultation with a named GP. This was different from the national GPPS where patients are asked to reflect on consultations that have occurred over the past 6 months, rather than a specific consultation. Our questionnaire contained 38 closed questions, 13 of which covered the socio-demographic profile of the patient and were not analysed in this study. The 25 questions covering the patients’ experience of care at the practice comprised 54 separate response items related to making an appointment (12), telephone access (4), access to a doctor (11), arriving at the appointment (6), continuity of care (3), opening hours (8), doctor-patient communication and trust (8), and overall satisfaction (2). Response options were categorical for 33 of these items and ordinal for the remaining 21.

### Sample size calculation

Calculations based on Fisher’s z-transformation of the intraclass correlation coefficient (ICC) showed that a sample of 250 completed re-test questionnaires would allow us to estimate ICCs for individual items with a 95 % margin of error of less than 0.1 for coefficients around 0.5 and less than 0.05 for coefficients of 0.8 or above. Based on response rates in initial pilot data of 38 questionnaires per doctor returned within 3 weeks and an estimated response rate of 75 % for the retest phase that we had observed in earlier study (Wright et al. 2012), we sought to recruit the patients from a minimum of nine doctors to this study.

### Data analysis

Data analysis was conducted using SPSS version 18 (SPSS 2009). We described the response rate and response timings for both test and retest phases and compared the demographic profiles (age, gender, ethnicity) of three groups of patients: those who were sent but did not return a test questionnaire within 3 weeks of mail out (and so were not eligible for the retest phase), those who were sent but did not return a retest questionnaire within 4 weeks of mail out, and those who returned both a test and a retest questionnaire within the deadlines. The proportions of non-response by patients eligible to answer each of the 54 separate response items were compared between the test and retest phases using Chi squared tests with a Holm-Bonferroni correction for multiple comparisons (Holm 1979). For the 33 categorical response items we measured test–retest reliability using raw agreement rates and Cohen’s Kappa statistic (Cohen 1968). For the 21 ordinal response items we assigned integer scores (1, 2, 3, etc.) to the meaningful response options (apart from any ‘Don’t know’ or ‘Not applicable’ options) and calculated ICCs. Both the ICCs and the Kappa statistics were interpreted as follows: <0.00 was poor, 0.00–0.20 was slight, 0.21–0.40 was fair, 0.41–0.60 was moderate, 0.61–0.8 was substantial and 0.81–1.00 was almost perfect (Landis and Koch 1977). We calculated the mean score on each item in the test and retest phases and investigated possible changes in the mean scores using paired sample t-tests, again with a Holm-Bonferroni correction.

## Results

20 doctors from five practices took part in the test–retest study. In the test phase we sent out questionnaires to 2877 patients who had recently consulted one of the participating GPs. Retest questionnaires were sent out to 597 patients who had returned a completed test questionnaire within 3 weeks of mail out. A total of 348 (58 %) patients returned a completed retest questionnaire within 4 weeks. Amongst those eligible for the retest phase the mean time from mail out to receipt of a completed questionnaire was 8.7 days in the test phase, and longer in the retest phase questionnaire (10.1 days). The demographic profile of patients, classified according to their level of participation in the study, is shown in Table 2. There were no gender differences between these groups, but retest responders tended to be older and this group contained more people of white British ethnicity.

**Table 2:** Demographic characteristics of patient sample by level of study participation with *P* value for tests of variation across the three groups

	Patients sent but not returning a test questionnaire within 3 weeks of mail out.^a^	Patients sent but not returning a retest questionnaire within 4 weeks of mail out.	Patients returning both a test and a retest questionnaire within the deadlines.	*P* value
Number	2009	249	348	n/a
Number (%) male	807 (40.1)	89 (35.7)	138 (39.7)	0.404
Number (%) white British^a^	404 (88.0)	204 (89.1)	326 (95.6)	0.001
Mean (SD) age in years	46.2 (18.5)	59.4 (18.8)	65.3 (15.1)	<0.001

^a^Ethnicity data was only available for those who returned a completed test questionnaire and responded to the ethnicity item. For patients sent but not returning a test questionnaire within 3 weeks of mail out, n = 473; for patients sent but not returning a retest questionnaire within 4 weeks of mail out, n = 229; for patients returning both a test and a retest questionnaire within the deadlines, n = 341

No significant differences in item non-response rates between the test and retest phase were found for any of the 54 items.

### Test–retest reliability of categorical items

The percentage agreement in responses to the 33 categorical items ranged from 66 to 100 % (mean 88 %), while the kappa coefficients ranged from 0.00 to 1.00 (mean 0.53) (Table 3). The raw agreement rates were 80 % or above for all but six of these items.

**Table 3:** Sample size, raw agreement (%) and Cohen’s kappa statistic for the 33 categorical items

Topic/item	N	Raw agreement (%)	Kappa
*Making an appointment*			
Q1a Normally book an appointment in person	348	82	0.63
Q1b Normally book an appointment by phone	348	95	0.69
Q1c Normally book an appointment by fax	348	100	1.00
Q1d Normally book an appointment online	348	99	0.93
Q1e Normally book an appointment by digital TV	348	100	^a^
Q1f Booking doesn’t apply	348	99	0.00
Q2a Prefer to book in person	348	81	0.62
Q2b Prefer to book by phone	348	85	0.44
Q2c Prefer to book by fax	348	99	0.50
Q2d Prefer to book online	348	93	0.79
Q2e Prefer to book by digital TV	348	100	^a^
Q2f No preference in booking an appointment	348	98	0.39
*Access to a doctor*			
Q4 In the past 6 months, have you tried to see the doctor quickly	334	82	0.49
Q5 Were you able to see the doctor quickly	234	83	0.46
Q6a If you couldn’t be seen quickly was this because there were no appointments	348	83	0.39
Q6b If you couldn’t be seen quickly was this because there the times did not suit you	348	97	0.46
Q6c If you couldn’t be seen quickly was this because the appointment was with a doctor you didn’t want to see	348	94	0.44
Q6d If you couldn’t be seen quickly was this because the appointment offered was with a nurse and you wanted to see a doctor	348	99	0.46
Q6e If you couldn’t be seen quickly was this because you were offered an appointment at a different branch	348	98	0.44
Q6f If you couldn’t be seen quickly was this because there was a different reason	347	98	0.43
Q6 g Can’t remember why you were unable to be seen quickly	348	97	0.43
Q7 In the past 6 months, have you tried to book ahead for an appointment with a doctor	339	79	0.44
Q8 Were you able to get an appointment with a doctor more than 2 weekdays ahead	239	73	0.40
*Arriving at the appointment*			
Q11 In the reception area, can other patients overhear what you say to the receptionist	339	80	0.59
*Continuity of care*			
Q15 Is there a particular doctor you prefer to see	338	91	0.68
Q17 Was your consultation with your preferred doctor	254	89	0.55
*Opening hours*			
Q19a As far as you know is the surgery open before 0800	330	75	0.46
Q19b As far as you know is the surgery open at lunchtime	309	71	0.49
Q19c As far as you know is the surgery open after 1830	307	66	0.47
Q19d As far as you know is the surgery open on Saturdays	309	80	0.42
Q19e As far as you know is the surgery open on Sundays	308	85	0.38
Q20 Would you like the surgery to be open at additional times	313	83	0.57
Q21 Which additional time would you most like your surgery to be open	111	77	0.49

^a^Left unticked by 100 % of respondents in both phases. Kappa cannot be calculated

### Test–retest reliability of ordinal response items

ICCs for the 21 ordinal items averaged 0.67 and ranged from 0.44 for question 9 *(*“How easy do you find it to get into the building at this GP surgery or health centre?”) to 0.77 for question 25 (“Would you recommend this GP surgery or health centre to someone who has just moved to your local area?”) (Table 4). The ICCs for all but one of these items (question 9) were above 0.6, representing substantial test–retest reliability. Compared to the test phase, mean scores in the retest phase rose for 8 and fell for 12 of the 21 items. After applying the Holm-Bonferroni procedure however, question 9 (relating to ease of access to premises) was the only item for which a significant difference was found between the mean scores in the test and retest phase (*P* = 0.001).

**Table 4:** Sample size, ICC (95 % confidence interval), mean test–retest difference (95 % confidence interval) and associated *P* value for the 21 ordinal response items

Topic/item	N	ICC (95 % CI)	Mean difference (95 % CI)	*P* value^a^
*Telephone access*				
Q3a How easy have you found getting through on the phone	333	0.73 (0.67, 0.78)	−2.40 (−4.91, 0.11)	0.061
Q3b How easy have you found speaking to a doctor on the phone	191	0.68 (0.59, 0.75)	−4.01 (−7.64, −0.39)	0.030
Q3c How easy have you found speaking to a nurse on the phone	82	0.63 (0.48, 0.75)	−2.85 (−8.62, 2.93)	0.330
Q3d How easy have you found getting test results on the phone	131	0.62 (0.51, 0.72)	0.25 (−3.88, 4.39)	0.903
*Arriving at the appointment*				
Q9 How easy do you find it to get into the building at this GP surgery or health centre?	345	0.44 (0.35, 0.52)	2.32 (0.94, 3.70)	0.001
Q10 How clean is this GP surgery or health centre?	344	0.60 (0.53, 0.66)	1.16 (−0.10, 2.42)	0.070
Q12 How helpful do you find the receptionists at this GP surgery or health centre?	335	0.69 (0.63, 0.74)	−0.60 (−2.39, 1.20)	0.514
Q13 How long after your appointment time do you normally wait to be seen?	315	0.67 (0.60, 0.73)	−0.95 (−2.60, 0.70)	0.257
Q14 How do you feel about how long you normally have to wait	308	0.70 (0.64, 0.75)	−2.11 (−4.43, 0.21)	0.074
*Continuity of care*				
Q 16 How often do you see the doctor you prefer	255	0.71 (0.64, 0.77)	−0.78 (−3.49, 1.92)	0.568
*Opening hours*				
Q18 How satisfied are you with the hours that this GP surgery or health centre is open?	325	0.65 (0.59, 0.71)	2.23 (0.40, 4.06)	0.017
*Doctor*-*patient communication and trust*				
Q22a How good was the doctor at giving you enough time	337	0.62 (0.55, 0.68)	0.45 (−0.96, 1.85)	0.532
Q22b How good was the doctor at asking about your symptoms	317	0.70 (0.64, 0.75)	−0.47 (−1.84, 0.90)	0.498
Q22c How good was the doctor at listening to you	331	0.72 (0.66, 0.77)	0.38 (−0.88, 1.63)	0.554
Q22d How good was the doctor at explaining tests and treatments	275	0.72 (0.65, 0.77)	−1.27 (−2.81, 0.26)	0.104
Q22e How good was the doctor at involving you in decisions about your care	275	0.68 (0.61, 0.73)	−1.00 (−2.65, 0.65)	0.233
Q22f How good was the doctor at treating you with care and concern	326	0.67 (0.61, 0.73)	0.23 (−1.16, 1.62)	0.745
Q22 g How good was the doctor at taking your problems seriously	324	0.72 (0.67, 0.77)	−0.08 (−1.46, 1.31)	0.913
Q23 Did you have confidence and trust in doctor you saw	340	0.70 (0.64, 0.75)	−0.15 (−1.86, 1.57)	0.866
*Overall satisfaction*				
Q24 In general how satisfied are you with the care you get at this surgery or health centre?	344	0.74 (0.69, 0.78)	−0.58 (−1.81, 0.65)	0.353
Q25 Would you recommend this GP surgery or health centre to someone who has just moved to your local area?	333	0.77 (0.73, 0.81)	0.00 (−1.51, 1.51)	1.000

^a^After applying the Holm-Bonferroni procedure with a family-wise Type I error rate of 5 %, only the *P* value for Q9 remains significant

## Discussion

### Summary

Overall, the test–retest reliability results of the survey items varied, with some items showing poor agreement over an interval of approximately 4 weeks between test and retest questionnaires (ease of access to the building), whilst others achieved excellent agreement (patients’ willingness to recommend their practice to a family member or friend).

A large majority of the ordinal items had high ICC values and high response agreements. Items relating to staff performance (such as helpfulness of receptionists, communication skills of GPs) achieved high stability over time. One possible reason for this might be that face to face interaction with staff, whether it is with receptionists or with health professionals within a consultation, has a lasting impact on a patient’s memory when compared with other experiences of the practice.

The categorical items achieved fair to almost perfect agreement, with the majority of the items demonstrating moderate to substantial agreement. Given that the kappa statistic is adversely affected by prevalence rates for dichotomous items (Feinstein and Cicchetti 1990), there is, we believe, a good case for giving greater weight to the raw agreement rates, which were observed to be high. Despite the mixed Kappa scores, the majority of the percentage agreements between the test and retest phases indicated good to excellent reliability of questions.

### Strengths and limitations

This is the first study to report on the stability of patient responses over time using items from the GP patient survey. The response rate for our retest phase was good, and similar to that observed in other test–retest exit surveys in primary care (Wright et al. 2012; Mead et al. 2008). Our sample was not fully representative of the wider patient population within England and Wales, thus a more diverse test–retest study involving patients from different ethnic and age groups needs to be conducted to fully understand the stability of responses from different patient groups over time. There are methodological limitations to the test–retest method. Karras (1997) suggested that one disadvantage of test–retest method is that the first administration of the questionnaire could influence the results of the second administration, in that the respondent could recall the answers provided at the test phase and replicate this for the retest phase (Karras 1997). Kimberlin and Winterstein highlight the trade-off between potential recall bias when the retest interval is short and the possibility that what you are measuring will have changed when the retest interval is large (Kimberlin and Winterstein 2008). In addition, patients could have attended further consultations with a doctor in the period between the test and retest phase which could have altered their responses on the retest questionnaire. We did not ask, or identify from practice records, whether or not respondents had visited their practice since the appointment that was specified in the covering letter accompanied with both the test and retest questionnaires, which should be consideration for future research. Due to design of the study focusing on specific consultations with named GPs, we were unable to replicate the exact timings of when the national GPPS questionnaires are distributed to patients following their consultations.

### Comparison with existing literature

Previous work has suggested that the timing of administering a questionnaire may have an impact on the patient’s reported satisfaction with a service (Crow et al. 2002; Allemann Iseli et al. 2014; Kong et al. 2007). Research addressing the issue of recall bias suggests that health status should be measured at short intervals, for example 1–2 weeks. (Kimberlin and Winterstein 2008; Patrick 1991). If the focus of the research is to recall specific events the time frame must of short duration and in the immediate past. Our study adopted a short interval, referring, as it did, to a consultation which had taken place within the past 3 weeks. A longer time interval between the patient’s consultation and receipt of a questionnaire may influence their recollection of the consultation (Selic et al. 2011; Sandberg et al. 2008). This brings into question the accuracy of reflections regarding a consultation which may be temporally remote. For example, the GP patient survey invites patients to comment on consultations which may have taken place up to 6 months previously.

Whilst some patient surveys used in the UK have been validated and psychometrically tested, test–retest reliability has not been reported for all surveys (Lockyer 2009). The questionnaire used in our study measured patients’ overall experience of their GP surgery, and incorporated items addressing GP communication skills, practice environment, access and overall satisfaction. The only questionnaires used widely within the UK and addressing a similar agenda are the General Practice Assessment Survey (GPAS) and Questionnaire (GPAQ) and the Improving Practice Questionnaire (IPQ), neither of which have the breadth of topic coverage seen in our survey. Test–retest reliability for GPAS was assessed when patients were asked to complete their test questionnaire at the practice following a consultation; the retest questionnaire was posted to them 1 week later, however the sample size used was considerably smaller in comparison to the sample within our study (Ramsay et al. 2000).

## Conclusions

This is the first test–retest study carried out on items derived from the national GP patient survey. Testing the stability of these particular GPPS items was important if GPs and policy makers want to assess how patient experience of primary care services has changed over time. Our findings indicate that most of the items considered within this survey have acceptable test–retest reliability across a short time interval, with items relating to staff achieving high reliability. The findings raise some concerns regarding the reliability of certain items in the survey in the time frame for which it was tested and have implications for the need to test the reliability of item responses over the longer time interval used in the national GPPS. Further research might usefully explore the performance of the national survey in more diverse samples across England and Wales, and across the longer time interval it encompasses.
